# Characterizing psychological states in professional athletes through EEG: sex-based differences

**DOI:** 10.17179/excli2024-7980

**Published:** 2025-01-03

**Authors:** Kittichai Tharawadeepimuk, Ampika Nanbancha, Ekarat Onnom

**Affiliations:** 1 College of Sports Science and Technology, Mahidol University, Nakhon Pathom, Thailand

**Keywords:** psychological states, quantitative EEG, electroencephalogram, theta and beta frequency bands, sex differences

## Abstract

The psychological states are essential to maintain a healthy balance and optimize performance, especially in athletes. Sex-related differences in elite athletes are one of the important factors, which are ambiguous. This study aims to explore sex differences in psychological states of 36 professional athletes (12M/24F, 21.6±3.9 y, 166.3±8.9 cm, 56.4±11.3 kg, mean±SD) in the lead-up to a major international competition using objective (quantitative electroencephalogram, QEEG) and subjective measures (Athletic Coping Skills Inventory, ASCI-28). Participants included fifteen gymnasts, eleven swimmers, and ten e-sports players. QEEG measurements were collected during 5 minutes at rest before athletes completed the ASCI-28 scale. Data were obtained during training camp within 3 months before the competition. QEEG data were analyzed using a generalized linear mixed modeling (GLMM) to report the linear predictor of QEEG in brain areas (random) and sex (fixed). A multivariate analysis of variance (MANOVA) was applied to ACSI-28 subscales data to compare sex groups. Female athletes exhibited higher EEG activity in the theta power in the parietal and occipital areas and the beta power in the pre-frontal and temporal areas. Furthermore, significant sex differences were found in the coping with adversity (*p* =0.038) and goal setting/mental preparation subscales of the ACSI-28. The correlations between the QEEG and questionnaire scores were low to moderate correlations for female athletes, and moderate correlations for male athletes. A significant correlation was observed in both male and female athletes between activity in the temporal region within theta and beta frequency bands (QEEG), and the coping with aversity subscale (questionnaire). These findings provide valuable insights for sports psychologists to design appropriate psychological interventions and for future studies examining the impact of differential psychological interventions for male and female athletes to optimize well-being and performance.

## Introduction

In the majority of cases, several gender differences in psychological state have been highlighted in the literature in responses to recovery after injury (Lisee et al., 2020[43]), motivation (Ahmed et al., 2020[1]), and anxiety and coping style (Ivaskevych et al., 2019[31]). The evidence suggests that men and women may respond differently to a range of psychological challenges. The most striking gender disparities in psychopathology are preserved across populations, of different ages or income levels. Women are diagnosed with depression and anxiety disorders roughly twice as often as men (Alonso et al., 2004[2]; Leray et al., 2011[40]; Parker and Hadzi‐Pavlovic, 2001[49]) and suffer from eating disorders 6 to 10 times more often than men (Preti et al., 2009[51]), while there is a notable male preponderance in externalizing disorders, such as alcohol and drug abuse (Alonso et al., 2004[2]; Hicks et al., 2007[27]; Lopez-Soler et al., 2009[44]). 

Although the psychological state is classically evaluated with questionnaires (Beckmann and Kellmann, 2003[4]), interviews (Huffcutt et al., 2001[30]), and behavioral assessment (Tkachuk et al., 2003[63]), more recent work suggests that electroencephalogram (EEG) has the potential to provide a more comprehensive understanding of cognitive and emotional status (Huang, 2021[29]; Li et al., 2021[41]). Indeed, analysis of EEG power showed gender-related differences in parietal lobe activation in response to depressing and sad music videos and limbic lobe activation in response to fun stimuli (Goshvarpour and Goshvarpour, 2019[21]), suggesting differences in brain networks for processing emotional stimuli. Moreover, during creative thinking, some authors proposed a different hemispheric organization between men and women which would be characterized by the beta2 power and coherence (Razumnikova, 2004[53]). Quantitative electroencephalogram (QEEG) is a neuroimaging technique that analyzes electrical activity in the brain to provide quantitative data on brainwave patterns, aiding in the assessment of psychological states and cognitive functioning. QEEG parameters also revealed gender differences during motor execution, with disparities highlighted in beta frequency band located in temporoparietal areas during simple hand movement tasks (Cantillo-Negrete et al., 2017[10]). In the sports context, athletes often find themselves in psychologically challenging situations. There were differences in sports interest, motivation, competitiveness, and responses to competition between men and women athletes. These differences in psychological factors can affect athletes' performance, training approaches, and responses to stressors in the sports environment (Deaner et al., 2016[16]).

Crucially, sports psychologists work closely with athletes to help them achieve and maintain peak performance while also addressing any mental barriers or challenges that may arise (Low et al., 2023[45]; Oudejans and Pijpers, 2009[48]). To do so, practitioners provide ongoing support, implement tailored mental training programs, and conduct thorough assessments (Harmison, 2006[26]; Wang and Zhang, 2015[65]). Assessing an athlete's psychological state is crucial as it provides valuable insights into various mental factors that can significantly impact athletic performance (MacNamara et al., 2010[46]; Schaal et al., 2011[57]). Moreover, understanding and managing the differences in psychological states is essential to maintain a healthy balance and sustain long-term well-being (Caruso et al., 1990[11]). 

Therefore, the main goal of this study was to explore the sex-related differences in the psychological state using QEEG frequency bands and the subscale of ACSI-28 among professional athletes in the lead-up to an international competition. We hypothesized that the psychological state of male and female athletes would be different with cognitive processing related to behavior performance (Ramos-Loyo et al., 2022[52]), which can be derived from the EEG theta frequency band (Kober and Neuper, 2011[38]). Additionally, we hypothesized that sex differences would be presented with executive functions (Gaillard et al., 2021[20]), typically associated with the EEG beta frequency band (Li et al., 2017[42]). The QEEG signal was characterized for six brain areas with corresponding electrodes which demonstrated both the functional and anatomical aspects of the brain's organization (Fan et al., 2015[18]; Strijkstra et al., 2003[60]). 

## Materials and Methods

### Participants

Thirty-six volunteer professional athletes (12M/24F, 21.6±3.9 y, 166.3±8.9 cm, 56.4±11.3 kg, mean±SD) consisted of fifteen gymnasts, eleven swimmers, and ten e-sports players. Thirty-one participants were right-leg and right-arm dominant, and five participants were left-leg and left-arm dominant. All of them were qualified to participate in the upcoming 31^st^ Southeast Asian Games. The participants provided written informed consent prior to their participation in this study. The experiments were approved and performed in accordance with the research ethics committee of Mahidol University - Central Institutional Review Board (COA No. MU-CIRB 2021/147.2206).

### Procedure and data collection

In this study, we used QEEG to examine the brain activity and Athletic Coping Skills Inventory-28 (ACSI-28) of professional athletes who would participate in the competition in the next three months. They were recruited and then informed about the purpose of the study, procedures, and potential risks before providing their written informed consent to participate. The experimental session started with the collection of QEEG measurements. Participants were then asked to complete the ACSI-28 questionnaire. 

The participants were familiarized with QEEG with demonstration and explanation of the equipment and procedure before the data collection. The participants were seated in a chair in a relaxed position. They were instructed not to move and to keep their eyes open. QEEG data were recorded for 5 minutes during a single recording. A recording was performed 3 months before the competition to establish the individual's QEEG.

### EEG measurements

An eego^TM^mylab ANT Neuro (32-electrodes EEG cap) was used in this study to record EEG signals at a sampling rate of 512 Hz and a notch filter of 50 Hz. A software filter was set to bandpass with a low pass of 0.3 Hz and a high pass of 100 Hz. The absolute power spectrum of the respective frequency bands derived by Fast Fourier Transformation (FFT) was expressed as follows: Delta (0.5-4 Hz), Theta (4.5-8 Hz), Alpha (8.5-13 Hz), and beta (13.5-30 Hz) wave ranges. The EEG instrument consisted of 32 channels at the recording of position and separated into 6 brain areas; Fp_1_, Fp_z_, and Fp_2_: pre-frontal; F_7_, F_3_, F_z_, F_4_, F_8_, FC_5_, FC_1_, FC_2_, and FC_6_: frontal; T_7_, and T_8_: temporal; C_3_, C_z_, C_4_, CP_5_, CP_1_, CP_2_, and CP_6_: central; P_3_, P_7_, P_z_, P_4_, P_8_, and PO_z_: parietal; O_1_, O_z_, and O_2_: occipital; CP_z_: reference electrode; GND: ground electrode. 

### ACSI-28 questionnaire

The Thai version of the ACSI-28 questionnaire was used to measure coping skills in sports (Kemarat, 2014[37]). The questionnaire consisted of 28 items measuring seven subscales including coping with adversity, peaking under pressure, goal setting/mental preparation, concentration, freedom from worry, confidence, and coachability. The participants were asked to respond to each item on a 4-point scale: 0 - almost never, 1 - sometimes, 2 - often, 3 - almost always. Each subscale consisted of four items that were averaged to provide a subscale range from 0 to 3. Additionally, the scales were then summed to yield a personal coping resource score. The original subscales, as reported by Smith et al. (1995[59]) were found to be internally consistent with alpha levels ranging from 0.62 to 0.78 and a total (personal coping resources) scale alpha of 0.86 (Smith et al., 1995[59]).

### Statistical analysis

In this study, there were quantitative EEG (QEEG) and Athletic Coping Skills Inventory (ACSI-28) data. Data normality was assessed using Shapiro-Wilk test, which indicated that all variables did not follow a normal distribution. Consequently, non-parametric statistic methods were employed for further analysis. A generalized linear mixed modeling (GLMM) was used to report the linear predictor between QEEG in brain areas (random) and sex (fixed) effects. Multivariate analysis of variance (MANOVA) was used to compare sex groups with respect to seven subscales of the ACSI-28 measuring. Effect sizes (*d*) are reported to determine the magnitude of difference between sex groups and assessed against Cohen's *d* definitions of: <0.2, 0.3, 0.5, and 0.8 for trivial, small, moderate, and large, respectively (Cohen, 2013[13]).

QEEG analysis yielded a total of 6,912 data points (36 participants x 32 electrodes x 6 brain areas) in each frequency domain for statistical analysis. Standard residual diagnostics were performed for the specification of the appropriate statistical model based on visual inspection of the residual plots. GLMM with a gamma distribution and inverse link function was fitted to the data for frequency domains to provide estimates of QEEG activity (using maximum likelihood and BOBYQA optimizer). The exception was for the delta frequency where an identity link was applied due to non-model convergence (i.e., poor fit). Sex and brain area were identified as fixed effects (formula: QEEG activity ~ Sex * Brain Area) and subject as a random effect (formula: ~1|Subject). Specifically, the model provided estimates of QEEG activity across 6 brain areas (pre-frontal, frontal, temporal, central, parietal, and occipital), enabling comparisons between males and females. Where significant main effects were identified, *post hoc* analysis was applied with a Holm correction. Back transformation of the inverse link function was subsequently performed to provide estimated marginal means for Sex * Brain areas interaction effects. Descriptive statistics including the mean and 95 % confidence intervals (95 % CI) are reported for all outcome variables. Statistical significance was accepted at the *p*<0.05 level. All statistical analyses were conducted within the R statistical framework (version 4.0, R Foundation for Statistical Computing). 

Quantitative data obtained with the ACSI-28 questionnaire were analyzed by using descriptive statistics. Multivariate analysis of variance (MANOVA) was used to compare sex groups with respect to seven subscales of the ACSI-28 measurements. Differences (*p* -values) of less than 0.05 were considered statistically significant.

Finally, we computed correlation analyses to explore the relationship between each QEEG band and the ACSI-28 questionnaire scores for each group. The level of statistical significance was set to 0.05 and we classified Spearman's rank correlation coefficients from 0 to 0.3 as negligible correlation, 0.3 to 0.5 as low, 0.5 to 0.7 as moderate, and 0.7 to 0.9 as high, 0.9 to 1.0 as very high (Mukaka, 2012[47]).

## Results

QEEG data were analyzed according to four frequency bands and six brain areas. ACSI-28 data were analyzed in seven subscales.

### QEEG data in frequency bands

**Figure 1:**
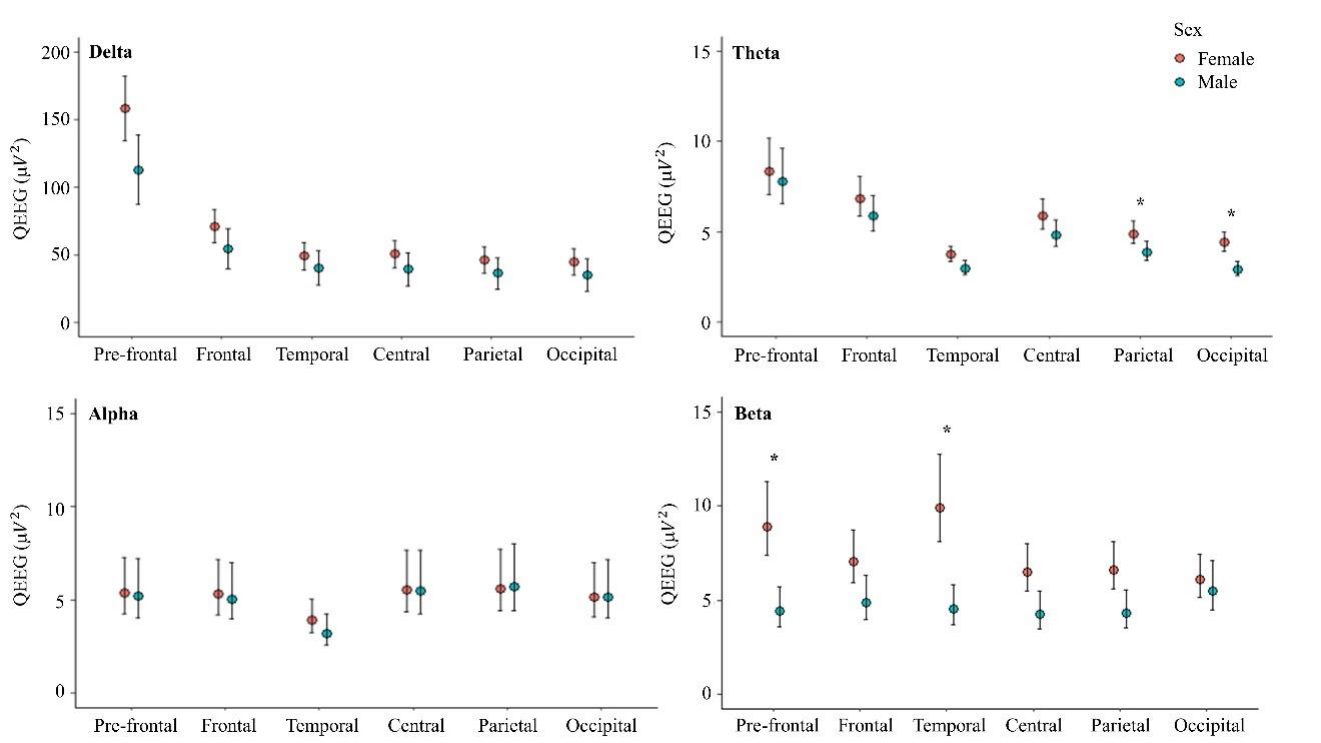
Descriptive statistics of QEEG data including the mean and 95 % confidence intervals (95 % CI). * Significance level seat at *p*<0.05.

QEEG data showed significant differences between male and female athletes in the theta and beta frequency bands. The descriptive statistics of QEEG data including the mean and 95 % confidence intervals (95 % CI) are shown in Figure 1(Fig. 1). In the theta frequency band, analysis of QEEG activity showed a significant interaction effect between sex and brain area (*p*<.001) as shown in Table 1(Tab. 1). QEEG activity in the parietal (P_3_, P_7_, P_z_, P_4_, P_8_, and PO_z_) and occipital areas (O_1_, O_z_, and O_2_) revealed higher QEEG's values in females *(p*=0.039 and *p*<0.001, respectively), with a large effect size (0.93 and 1.27).

In the beta frequency band, analysis of QEEG activity showed a significant interaction effect between sex and brain area (*p*=0.024) as shown in Table 1(Tab. 1). Beta QEEG activity in the pre-frontal (Fp_1_, Fp_z_, and Fp_2_) and temporal area (T_7_ and T_8_) revealed higher QEEG's values in female athletes (*p*<.001) with a large effect size (1.37 and 0.81, respectively).

### Questionnaire data

**Figure 2:**
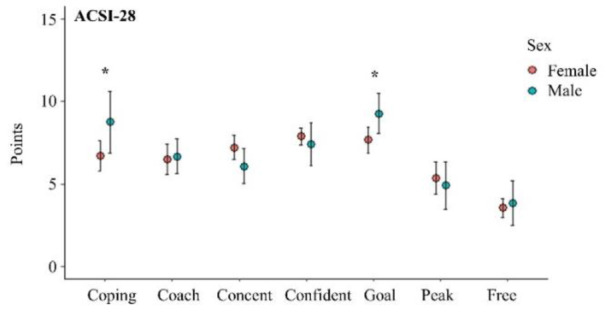
Descriptive statistics of ACSI-28 data including the mean and 95 % confidence intervals (95 % CI). *Significance level seat at *p*<0.05. (Abbreviation: Coping = Coping with adversity, Coach = Coachability, Concent = Concentration, Confident = Confidence and achievement motivation, Goal = Goal setting/Mental Preparation, Peak = Peaking under pressure, Free = Freedom from worry)

As shown in Table 2(Tab. 2), examination of the multiple regression analyses indicated different scores in the coping with adversity and goal setting/mental preparation subscales between male and female athletes (*p*=0.038 and *p*=0.035, respectively). Male athletes scored significantly higher on coping with adversity and goal setting/mental preparation. The descriptive statistics of ACSI-28 data including the mean and 95 % confidence intervals (95 % CI) is shown in Figure 2(Fig. 2). 

### Correlation between QEEG and questionnaire data

The correlation analysis between the QEEG frequency bands and subscales of the ACSI-28 questionnaire scores is summarized in Tables 3(Tab. 3) and 4(Tab. 4). For female athletes, the QEEG of delta, theta, and beta frequency bands showed significant correlations with the ACSI-28 coping with adversity, goal setting/mental preparation, peaking under pressure, and freedom from worry subscales. Regarding the male athletes, the QEEG of delta, alpha and beta frequency bands were significantly correlated with the coping with adversity, concentration, confidence and achievement motivation, and freedom from worry subscales. The obtained correlations between the QEEG and questionnaire scores (Tables 3-4(Tab. 3)(Tab. 4)) were low to moderate correlations for female athletes, and moderate correlations for male athletes (Mukaka, 2012[47]).

See also the supplementary data.

## Discussion

The objective of this study was to explore the sex-related differences in psychological states in athletes in the lead up to a competition using QEEG frequency bands and the ACSI-28 questionnaire. Our findings show significant differences between male and female athletes in the QEEG theta and beta frequency bands, and in the coping with adversity and goal setting/mental preparation subscales of the ACSI-28. 

### Sex difference in QEEG data 

We found higher QEEG values for females compared to male athletes in the theta frequency band in both the parietal and occipital areas (Figure 1(Fig. 1) and Table 1(Tab. 1)). These findings could indicate that female athletes had higher activation in areas of the brain related to cognitive processing (Sahar et al., 2022[55]; Wada et al., 1994[64]). Theta power has been used in numerous studies to explain the function correlated with human behavior (Billeke et al., 2014[5]; Bush et al., 2017[8]; Kahana, 2006[33]). Indeed, evidence indicates that theta power is associated with a range of cognitive affective-processes including sensorimotor processing, processing, and various attention-related mechanism such as arousal, passive attention (preattention), facilitatory active attention (selective attention, focus attention) (Karakaş, 2020[36]). Specifically, it was reported that theta power within cognitive control neural networks plays a key role in individual differences in cognitive goal-setting and strategy (Cooper et al., 2017[14]). The elevated theta band activity observed in female athletes may suggest enhanced cognitive control, particular in aspects of focused attention, goal-setting and strategy. The heightened theta activity in frontal or temporal brain areas may provide value insights into mental workload, arousal levels, and cognitive control mechanisms that could potentially differ between sexes within the athlete cohort. Interestingly, the previous study (Kober and Neuper, 2011[38]) reported no significant differences in cortical theta rhythm between sexes during resting period; however, the differences appeared during cognitive task performance. In contrast, our findings reveal sex-based differences in theta power during resting state. A key explanation for this discrepancy may lie in our recruited participants were elite athletes, who likely possess distinct personality and characteristics that could influence cognitive processing. Nonetheless, our findings highlight that sex-related differences persist within the athlete cohort, potentially indicating unique cognitive or neural adaptations in this population. 

Another sex difference was demonstrated in the QEEG activity of athletes in the beta frequency band in the pre-frontal and temporal areas, with higher values found in female athletes (Figure 1(Fig. 1) and Table 1(Tab. 1)). This enhancement is related to executive functions (Bočková et al., 2007[6]; Wang et al., 2023[66]), particularly coping skills and executive function, which are associated with activity in the right dorsomedial prefrontal cortex (Bradshaw et al., 2017[7]). Additionally, some researchers have proposed that an increase in beta power might serve as a general indicator of inhibition across voracious cognitive domains, including motor control, perception, and memory (Castiglione et al., 2019[12]; Pastötter et al., 2008[50]; Tempel et al., 2020[62]). The changes of beta frequency band in the pre-frontal and temporal areas could be influenced by experiences that promote the maturity of the prefrontal cortex, particularly through interaction with the environment (Sastre-Riba, 2006[56]). This could possibly explain an increased beta value found in female athletes in the present study. Our finding is consistent with the work of Aurlien et al. and Jaušovec and Jaušovec, who reported that women exhibited a higher EEG beta power than men under resting conditions (Aurlien et al., 2004[3]; Jaušovec and Jaušovec, 2010[32]). Moreover, this agreed with findings from studies that observed similar results in simple visual stimulus (Güntekin and Başar, 2007[23]) and mental rotation tasks (Butler et al., 2006[9]; Rescher and Rappelsberger, 1999[54]), where female athletes in our study demonstrated higher beta frequency values, despite differences in the specific measurement tasks used. The increase in beta frequency band observed in female participants is also evident in elite athletes, as reported in our study. Significant sex differences in brain activity, as well as in behavioral measures, have been documented, suggesting that athletes exhibit unique neurophysiology characteristics compared to non-athletes (Fang et al., 2022[19]). However, even within the athlete population, differences between sexes persist, particular in theta and beta frequency bands.

### Sex difference in questionnaire data

The coping skill results from the ACSI-28 assessment among male and female athletes showed differences in subscales of coping with adversity, and goal setting/mental preparation (Figure 2(Fig. 2) and Table 2(Tab. 2)). Significant differences between sex in psychological aspects of athletic performance have been documented (Schmidt et al., 2019[58]). Sex could have an impact on individuals' effectiveness in coping stress. It has been reported that males and females differ in their coping strategies. Specifically, within the emotion-focused coping strategies, females demonstrate a significantly greater need for emotional engagement compared to males (Tamres et al., 2002[61]). Additionally, male and female athletes may use different coping skills when dealing with stressful situations (Kaiseler et al., 2012[34]). This may help explain our findings, which similarly demonstrate sex differences in coping strategies. Regarding subscale of goal setting/mental preparation, which is closely linked to achievement-related cognition and behavior in sports (Kaplan and Flum, 2010[35]), our results reveal that males scored higher on this subscale. This could suggest that male athletes may be more effective at establishing concrete goal, maintaining focus and, employing mental strategies to enhance readiness and resilience before competition (Gould and Maynard, 2009[22]). This finding aligns with the study of Koh and Wang, which indicated that male athletes generally exhibit more competitive tendencies and set higher performance goals than female athletes (Koh and Wang, 2015[39]). In addition, the observed sex differences may stem from the distinct focus of the competition, as this can influence individual achievement goal orientation (Hanrahan and Cerin, 2009[24]).

The results of ACSI-28 questionnaire, which assess coping skills, appear consistent with our QEEG finding, which demonstrates differences in theta and beta frequency bands. This alignment perhaps reflects a connection between our findings in both brain and behavior. Specifically, the increased amplitude of theta and beta frequency bands in female athletes suggests that females might need recruit greater neural activity to respond effectively to in cognitive demand (Engel and Fries, 2010[17]; Hanslmayr et al., 2012[25]). It is recognized that psychological factors are crucial to athletic performance. Therefore, studying sex differences in psychological aspects provides valuable insight into their influence on performance in competitive sports. These differences should be taken into account when addressing with male or female participation in sports, as they can influence various aspects of performance and psychological response. The results of the present study revealed that male and female athletes had different psychological responses in both brain and behavior. Therefore, this finding also revealed psychological state of athletes and confirmed the significant role of sex in coping strategies with competition-related stress. Importantly, in sports, coping variations are inconclusive and critical. There is still much to know about the differences in coping in sports. This could help bridge this research gap and/or inconsistencies currently dominating coping literature.

### Correlation of QEEG and questionnaire score (separate analyses for male and female athletes)

The correlation analysis revealed low to moderate correlations between QEEG and subscales of the ACSI-28 questionnaire (Table 3(Tab. 3)). Given the primary focus on significant sex difference in this study, the analyses were conducted separately for male and female groups to examine the correlations between brain activity and questionnaire scores within each group. Consequently, our findings underscore the significance of coping with adversity and goal setting/mental preparation subscales, and highlighted theta and beta frequency bands in relation to brain activity. Specifically, we found correlations involving theta frequency band in female athletes, with associations between coping with adversity and goal setting/mental preparation subscales in temporal and frontal areas. These results align with findings in male athletes, where correlations were observed in beta frequency band. This suggests that trait coping skills may influence brain networks particularly increased temporal lobe activity associated with active coping, as observed in the prior study (Jaušovec and Jaušovec, 2010[32]). Thus, our findings provide evidence of link between QEEG measure and the ACSI-28 questionnaire in relation to coping skills. The absence of significant correlations between other QEEG parameters and additional ACSI-28 subscales could be distributed to the varied coping strategies employed by athletes (Corsi-Cabrera et al., 2007[15]), as well as to differences in cognitive processing stages, with involuntary attention in QEEG recording contrasting with the voluntary cognitive response required in questionnaire completion (Huang et al., 2020[28])

### Limitations of the study

The present study has some limitations. First, the small sample size used for the correlation analysis is one of the limitations. A larger or smaller sample size could have produced different results. Additionally, we only measured the resting state of the brain activity by QEEG to evaluate the psychological state of athletes. However, if we had included another task in the test, the brain's reaction might have been even more detailed.

## Conclusions

This study highlighted sex differences among elite athletes residing in sports camps prior to the crucial competition event. QEEG testing during the resting state revealed higher activation in females compared to male athletes in theta frequency band at the parietal and occipital areas, and the beta frequency band at the pre-frontal and temporal areas. The functions of these brain areas are associated with cognitive processing and executive functions. Additionally, ACSI-28 measurements demonstrated significant sex differences in coping with adversity and goal setting/mental preparation subscales, with male athletes scoring higher than female athletes. These findings suggest that male athletes might be more effective at setting concrete goals, maintaining focus, and using mental strategies to enhance readiness and resilience before competition. The observed sex differences were notably evident in the theta and beta frequency bands and related to individual characteristics such as coping and goal setting/mental preparation. These findings provide useful insights for sports psychologists in designing targeted psychological interventions aimed at optimizing athletes' peak performance in competitions. 

## Declaration

### Data availability statement 

The dataset supporting the findings of this study is available from the corresponding author upon reasonable request.

### Ethics declaration 

The study was conducted in accordance with the Declaration of Helsinki, and approved by the research ethics committee of Mahidol University - Central Institutional Review Board (protocol code COA No. MU-CIRB 2021/147.2206 and approved on 22 June 2021).

### Authorship contribution statement

KT conceptualized and designed the study, submitted the project to the ethics committee, contributed to data collection, performed formal data analysis, interpreted the findings, and drafted and finalized the manuscript; EO conceptualized and designed the study, recruited participants, contributed to data collection, performed data analysis, interpreted the findings, and approved the final version of the manuscript; AN participated in the study design, recruited participants, contributed to data collection, and approved the final version of the manuscript. All authors reviewed and contributed to the final manuscript. All authors have read and approved the final version of the manuscript and agree with the order of presentation of the authors.

### Acknowledgments

We thank Dr. Frederic Stucky for his suggestions in the preparation of this manuscript. This work was supported by College of Sports Science and Technology, Mahidol University, Thailand.

### Declaration of competing interest 

The authors declare that they have no known competing financial interests or personal relationships that could have appeared to influence the work reported in this paper.

## Supplementary Material

Supplementary data

## Figures and Tables

**Table 1:**
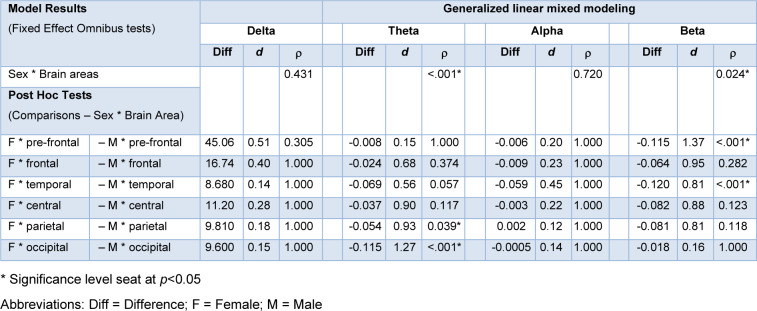
Generalized linear mixed modeling (GLMM) of QEEG in brain areas and sex

**Table 2:**
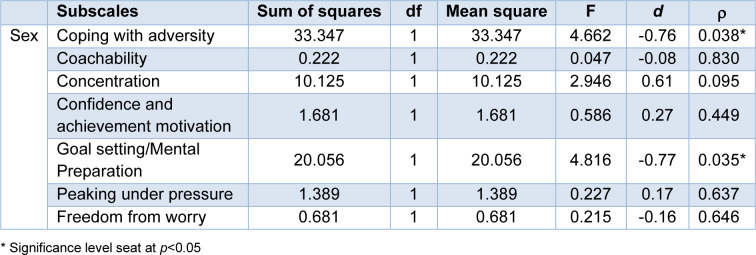
Analysis of the ACSI-28 subscales for depending on sex and MANOVA results

**Table 3:**
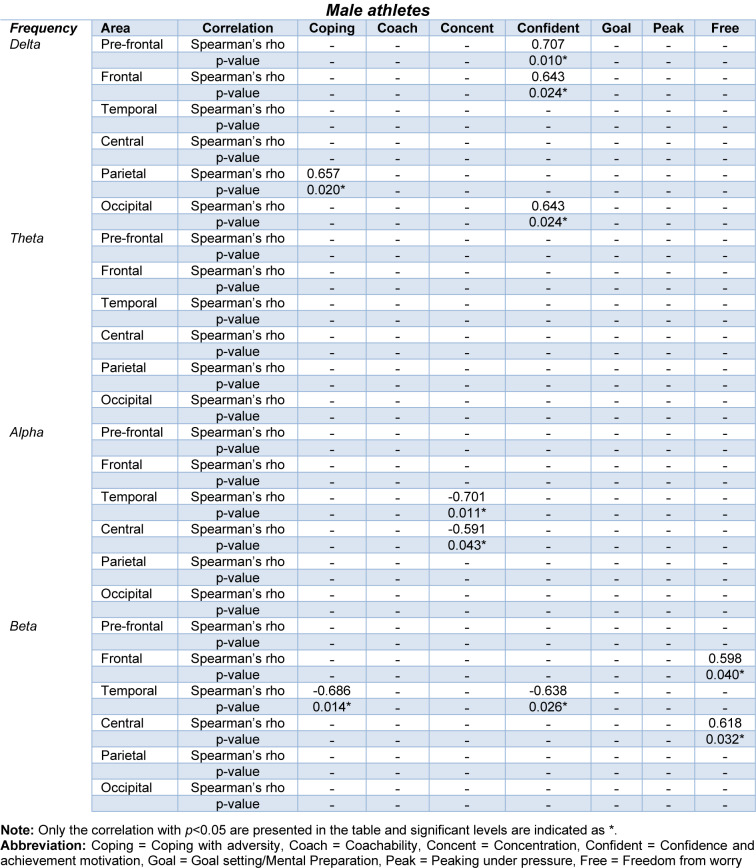
Summary of correlations between QEEG and subscales of the ACSI-28 questionnaire (Male)

**Table 4:**
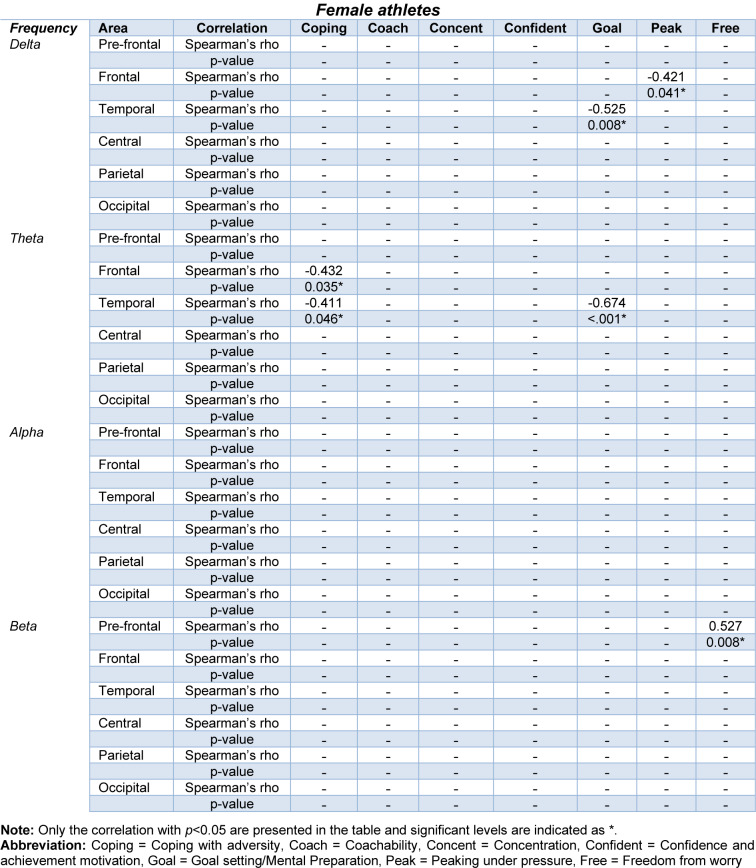
Summary of correlations between QEEG and subscales of the ACSI-28 questionnaire (Female)
