# Supplementary material for: Characterizing psychological states in professional athletes through EEG: sex-based differences
Source: EXCLI J. 2025 Jan 3;24:1–14. doi: 10.17179/excli2024-7980 (PMC11847956; doi:10.17179/excli2024-7980)
Supplement: Supplementary data [file EXCLI-24-1-s-001.pdf]

EXCLI Journal 2025;24:1-14 – ISSN 1611-2156
Received: October 29, 2024, accepted: November 19, 2024, published: January 03, 2025

Supplementary data to:

Original article:

CHARACTERIZING PSYCHOLOGICAL STATES IN PROFESSIONAL ATHLETES THROUGH EEG: SEX-BASED DIFFERENCES

Kittichai Tharawadeepimuk, Ampika Nanbancha, Ekarat Onnom*

College of Sports Science and Technology, Mahidol University, Nakhon Pathom, Thailand

* **Corresponding author:** Dr. Ekarat Onnom, College of Sports Science and Technology, Mahidol University, 999 Phuttamonthon 4 Road, Salaya, Nakhon Pathom, 73170 Thailand. Tel. +662-441-4295-8. E-mail: ekarat.onn@mahidol.edu

https://dx.doi.org/10.17179/excli2024-7980

This is an Open Access article distributed under the terms of the Creative Commons Attribution License (http://creativecommons.org/licenses/by/4.0/).

EXCLI Journal 2025;24:1-14 – ISSN 1611-2156
Received: October 29, 2024, accepted: November 19, 2024, published: January 03, 2025

Table I: Descriptive characteristics of the participants

ID	Type of Sports	Sex	Weight (kg)	Height (cm)	Age (years)	Dominant
**1**	gymnast	F	56	162	24	R
**2**	gymnast	F	55	168	24	R
**3**	gymnast	F	59	160	22	R
**4**	gymnast	F	54	162	24	R
**5**	gymnast	F	46	154	18	R
**6**	gymnast	F	47	170	18	R
**7**	gymnast	F	50	168	18	R
**8**	gymnast	F	47	155	18	R
**9**	gymnast	F	52	159	20	R
**10**	gymnast	F	49	157	18	R
**11**	gymnast	F	50	158	21	R
**12**	gymnast	F	51	170	18	L
**13**	gymnast	M	64	164	24	R
**14**	gymnast	M	65	168	20	L
**15**	gymnast	M	55	164	20	R
**16**	swimmer	M	77	185	20	R
**17**	swimmer	F	58	166	36	R
**18**	swimmer	M	72	180	21	R
**19**	swimmer	F	57	163	18	L
**20**	swimmer	M	94	193	28	R
**21**	swimmer	F	49	160	26	R
**22**	swimmer	F	60	173	25	R
**23**	swimmer	M	75	178	18	R
**24**	swimmer	F	49	160	18	L
**25**	swimmer	F	63	164	18	R
**26**	swimmer	F	53	164	18	R
**27**	e-sports player	F	40	165	27	R
**28**	e-sports player	M	53	171	24	R
**29**	e-sports player	M	45	167	26	R
**30**	e-sports player	M	50	170	23	R
**31**	e-sports player	F	53	160	25	R
**32**	e-sports player	F	46	160	22	R
**33**	e-sports player	F	55	155	21	R
**34**	e-sports player	F	47	159	22	R
**35**	e-sports player	M	53	185	18	L
**36**	e-sports player	M	80	168	18	R

Abbreviations: F = Female; M = Male; R = Right; L = Left

Table II: QEEG data within the delta frequency band for all participants

ID	Brain areas (μV^2)					
	**Pre-frontal**	**Frontal**	**Temporal**	**Central**	**Parietal**	**Occipital**
**1**	141.94	38.648	34.141	23.304	23.569	22.973
**2**	90.122	39.245	22.759	25.967	26.222	23.480
**3**	85.066	48.055	30.220	41.539	36.794	34.992
**4**	75.671	42.442	55.719	30.473	32.671	44.469
**5**	195.56	78.222	37.489	52.980	42.976	36.527
**6**	176.53	102.37	61.603	74.334	60.664	61.073
**7**	101.64	33.946	13.336	24.412	19.749	19.290
**8**	201.63	81.160	49.219	55.449	47.569	39.333
**9**	187.48	53.376	36.900	44.608	39.302	32.131
**10**	133.34	79.851	46.783	52.965	47.748	45.336
**11**	113.68	59.622	31.199	40.159	31.778	22.314
**12**	138.68	55.713	32.472	34.051	27.882	30.055
**13**	109.82	56.843	36.761	36.548	29.136	21.525
**14**	65.103	31.565	19.608	25.288	21.523	18.229
**15**	107.41	45.396	25.056	28.541	27.734	30.589
**16**	107.75	59.123	58.929	47.107	50.872	27.162
**17**	331.13	84.388	55.266	55.957	56.358	74.323
**18**	133.02	70.010	38.144	34.811	26.302	33.718
**19**	112.49	89.600	51.383	71.589	67.346	73.822
**20**	58.655	33.096	18.162	42.694	33.706	33.548
**21**	81.940	59.746	49.931	35.122	34.336	32.634
**22**	235.07	150.00	159.48	94.147	92.971	95.281
**23**	85.326	49.450	40.604	31.450	26.394	16.008
**24**	230.95	77.300	25.831	34.534	27.465	21.861
**25**	162.74	95.816	115.16	60.477	56.478	57.114
**26**	128.29	96.300	79.294	95.999	93.611	79.989
**27**	121.93	50.500	25.664	25.459	20.467	19.460
**28**	113.37	71.824	61.496	61.199	60.515	57.150
**29**	277.36	88.228	103.12	45.940	55.818	68.207
**30**	230.05	109.81	88.850	84.971	79.508	83.703
**31**	150.25	65.200	36.656	37.031	30.918	21.759
**32**	403.95	176.00	115.91	127.95	91.486	97.007
**33**	148.31	61.200	46.434	48.002	41.642	43.382
**34**	97.288	34.400	13.924	18.405	24.232	15.512
**35**	105.73	64.272	31.329	39.553	43.685	65.509
**36**	67.740	47.558	38.537	45.545	37.697	24.626

EXCLI Journal 2025;24:1-14 – ISSN 1611-2156
Received: October 29, 2024, accepted: November 19, 2024, published: January 03, 2025

Table III: QEEG data within the theta frequency band for all participants

ID	Brain areas (μV^2)					
	**Pre-frontal**	**Frontal**	**Temporal**	**Central**	**Parietal**	**Occipital**
**1**	4.4520	4.2705	1.8664	3.9654	3.1112	2.3790
**2**	9.1354	7.9667	3.1333	7.9711	7.1573	5.3365
**3**	6.3612	7.6386	3.2213	7.1465	5.5607	4.4954
**4**	5.2480	5.4982	3.9216	5.4466	5.1271	6.1572
**5**	13.448	9.1063	4.7353	8.2002	7.4696	6.0638
**6**	12.185	8.8432	4.4439	7.5630	6.2253	7.0284
**7**	7.1208	7.1221	2.1465	5.6780	3.5206	2.8431
**8**	11.939	9.7358	4.3311	7.8034	5.6765	6.4728
**9**	8.7740	6.5191	3.0906	5.7488	4.4290	3.1063
**10**	8.8178	7.8757	4.2392	7.1515	5.3331	4.4794
**11**	9.5662	6.5335	2.5365	5.0447	4.1563	3.0277
**12**	9.4419	9.1293	6.0137	8.1080	6.7203	6.2032
**13**	6.5484	5.2960	2.3483	4.3825	3.6383	3.3124
**14**	5.9197	4.6531	2.2569	3.7586	2.8622	2.1136
**15**	7.7804	6.3103	2.7239	4.5746	3.5465	3.4446
**16**	12.214	7.6737	4.4741	6.4016	5.2643	2.7468
**17**	8.1293	7.0962	3.9614	7.3447	6.5406	6.3468
**18**	10.642	7.7488	3.9504	6.6177	5.6959	3.5089
**19**	5.9068	5.7100	2.5321	4.8262	4.8702	6.1648
**20**	8.9607	8.5558	3.6466	5.8822	5.3791	4.6826
**21**	5.4786	5.5949	3.0703	3.4783	2.8189	2.4794
**22**	10.911	11.000	10.097	7.7767	6.5794	6.1700
**23**	4.6924	4.5696	2.4056	3.8972	2.6071	1.2826
**24**	18.380	8.8000	2.9599	5.5399	4.4994	2.9718
**25**	8.9823	7.2461	3.5742	6.3548	4.7547	4.9036
**26**	10.640	6.5600	2.9285	5.3696	4.5673	4.1423
**27**	8.9506	6.6200	3.2760	4.9716	4.1129	4.0404
**28**	6.8918	5.6922	3.5835	4.8141	4.0408	3.3592
**29**	20.611	6.9988	3.1565	4.5186	3.0616	2.5730
**30**	9.9091	6.2039	2.3789	4.8158	3.2786	2.4200
**31**	13.199	10.500	5.0560	8.3805	6.6155	4.2245
**32**	20.750	9.9000	4.8390	7.1318	5.7223	4.9936
**33**	6.7578	6.0300	3.5222	5.2746	4.1424	4.1973
**34**	9.3760	7.1700	4.1788	7.2733	5.9455	3.9769
**35**	9.3234	8.2428	3.5565	5.9282	5.0742	4.3317
**36**	6.2421	6.0448	2.6949	6.8609	4.8237	2.8419

EXCLI Journal 2025;24:1-14 – ISSN 1611-2156
Received: October 29, 2024, accepted: November 19, 2024, published: January 03, 2025

Table IV: QEEG data within the alpha frequency band for all participants

ID	Brain areas (μV^2)					
	**Pre-frontal**	**Frontal**	**Temporal**	**Central**	**Parietal**	**Occipital**
**1**	3.1000	2.7056	1.7097	2.4924	2.1890	1.7395
**2**	10.144	12.184	4.9061	20.973	22.105	9.6928
**3**	4.2361	4.7081	2.3478	6.4573	8.5794	7.2543
**4**	5.2003	5.5237	3.1050	7.3234	9.3489	6.7734
**5**	19.227	22.384	12.856	26.935	26.788	19.802
**6**	9.0407	8.6336	5.1608	14.437	10.289	8.6636
**7**	12.578	10.683	5.2133	18.397	20.502	13.976
**8**	6.4629	5.5840	4.0604	6.3832	5.7610	5.1551
**9**	4.8917	4.8318	2.9628	4.3544	3.7747	3.1686
**10**	26.315	23.908	11.575	24.558	23.921	22.795
**11**	6.9096	6.9456	4.7693	6.8017	7.2189	4.5476
**12**	11.578	10.848	8.7864	10.832	10.950	8.8839
**13**	3.5622	3.0471	1.3678	3.0460	2.5137	2.0194
**14**	3.4114	3.1775	2.0335	2.8695	2.2874	1.9614
**15**	8.2052	9.3822	5.6232	17.238	16.238	10.573
**16**	4.7449	5.3182	3.3972	8.7675	7.2961	3.4227
**17**	10.794	10.507	4.6185	12.607	19.696	18.879
**18**	40.395	35.330	13.285	32.477	42.890	34.791
**19**	3.7267	3.6500	2.6792	5.7677	4.9226	4.1554
**20**	3.4261	3.0277	2.3568	3.5290	5.5011	9.0996
**21**	1.9552	2.2886	1.6339	1.7318	1.6731	1.5304
**22**	4.0735	4.6200	6.1546	3.6388	3.3970	3.4859
**23**	3.8544	4.8242	3.1020	8.1927	7.7198	2.6751
**24**	11.917	10.500	4.0613	8.0666	8.0675	6.3919
**25**	4.6874	4.6705	2.6201	5.8750	5.2562	4.9447
**26**	6.4427	4.5700	3.2166	7.5124	6.3655	8.3370
**27**	4.8887	4.0400	4.3275	3.4911	3.7939	3.8075
**28**	3.2163	2.5213	1.8064	2.2172	2.0151	1.9585
**29**	4.4152	2.4966	1.7617	2.1329	1.9743	1.4708
**30**	6.9981	6.3318	3.0669	7.4225	10.332	8.6546
**31**	35.405	33.700	15.999	27.623	29.450	21.781
**32**	5.4738	4.0700	2.7500	3.9361	3.9639	5.7044
**33**	9.9346	11.000	5.7178	11.760	10.932	10.357
**34**	5.8691	5.0400	3.2180	6.8817	5.8820	4.1698
**35**	4.0906	3.8749	1.7773	3.1973	2.8773	2.5601
**36**	5.6082	7.2108	3.7717	11.738	12.039	11.338

EXCLI Journal 2025;24:1-14 – ISSN 1611-2156
Received: October 29, 2024, accepted: November 19, 2024, published: January 03, 2025

Table V: QEEG data within the beta frequency band for all participants

ID	Brain areas (μV^2)					
	**Pre-frontal**	**Frontal**	**Temporal**	**Central**	**Parietal**	**Occipital**
**1**	7.9054	5.2861	18.452	4.3301	3.7458	3.0946
**2**	10.093	7.6612	17.440	5.5663	5.9524	4.0422
**3**	5.8408	4.1142	2.6264	3.1804	3.5378	2.9675
**4**	5.5535	5.2829	4.2204	5.7208	8.5386	7.4166
**5**	9.8824	9.3236	50.440	8.6048	8.2558	6.2599
**6**	8.8630	9.1669	8.8444	8.8990	8.0903	7.3227
**7**	21.182	7.0361	3.7957	7.2040	6.1261	6.8634
**8**	14.795	10.512	15.347	10.260	10.393	9.0719
**9**	5.0857	5.4092	4.5281	5.3470	5.0390	3.9792
**10**	6.3000	6.4200	4.1571	6.6777	5.7066	5.5835
**11**	8.0929	8.5579	16.722	8.0869	8.5876	6.0283
**12**	15.600	8.7247	12.357	5.3097	5.1895	5.8715
**13**	5.2487	4.9498	2.0438	4.5275	3.6483	2.5695
**14**	4.6847	4.4446	9.9424	4.2762	4.0468	4.2040
**15**	3.9695	3.8977	2.2718	3.9902	4.1416	4.1665
**16**	6.1396	6.7051	5.9682	6.0744	6.4994	5.8306
**17**	16.023	14.761	17.682	16.027	14.631	11.943
**18**	5.2138	4.7137	6.3599	4.6350	5.8682	6.7458
**19**	11.087	8.3400	6.8326	8.4248	8.0685	6.0650
**20**	3.8686	6.7013	7.4421	5.4122	5.7172	14.589
**21**	6.7732	6.5567	6.2356	3.1068	2.6355	2.3984
**22**	7.8326	7.5200	16.878	6.1592	5.5693	4.5987
**23**	3.6308	4.7413	5.0384	3.4285	3.5622	3.1197
**24**	11.282	12.000	7.7270	8.6150	8.3974	6.7360
**25**	12.960	5.9674	5.3444	4.6706	5.2769	8.7188
**26**	3.9086	3.3100	4.2682	3.2878	4.3746	11.950
**27**	16.129	15.100	31.447	14.892	18.124	11.649
**28**	4.2017	2.8395	4.2303	2.4443	2.3798	2.8497
**29**	4.4029	3.1653	2.4969	2.6823	2.3751	1.8563
**30**	5.1663	6.7938	4.9369	8.0417	6.3878	4.6784
**31**	11.045	11.400	20.108	12.650	14.851	8.8038
**32**	7.2284	6.2600	4.5935	5.6281	5.5314	7.4142
**33**	5.9458	5.9100	4.2274	5.5153	4.6018	4.8886
**34**	24.047	4.5800	8.7347	3.7572	3.4293	4.8026
**35**	5.9831	6.2169	3.6438	4.6577	3.9247	3.6778
**36**	4.2035	8.3211	3.9212	4.3700	6.6394	18.105

EXCLI Journal 2025;24:1-14 – ISSN 1611-2156
Received: October 29, 2024, accepted: November 19, 2024, published: January 03, 2025

Table VI: Athletic Coping Skills Inventory-28 (ACSI-28) data for all participants

ID	ACSI-28 subscales (points)						
	**Coping**	**Coach**	**Concent**	**Confiden**	**Goal**	**Peak**	**Free**
**1**	9	9	9	8	9	7	3
**2**	10	8	8	11	10	7	5
**3**	9	7	9	8	11	6	2
**4**	7	6	6	9	7	6	2
**5**	11	8	12	9	9	12	4
**6**	7	10	9	7	9	4	3
**7**	8	6	6	6	10	4	5
**8**	6	3	7	8	6	2	5
**9**	6	4	9	8	7	6	3
**10**	9	9	8	8	9	6	5
**11**	5	6	7	6	9	8	2
**12**	5	5	7	7	5	3	4
**13**	11	9	8	9	11	8	4
**14**	5	5	5	2	7	5	4
**15**	9	7	7	8	9	5	1
**16**	5	6	4	6	8	1	3
**17**	10	10	8	9	8	6	4
**18**	5	3	5	8	9	3	3
**19**	8	10	7	7	8	7	6
**20**	7	9	7	8	6	4	2
**21**	7	8	7	8	5	5	4
**22**	3	7	3	5	3	5	1
**23**	4	6	3	6	8	1	3
**24**	5	6	5	8	8	3	4
**25**	4	3	5	6	8	1	3
**26**	5	6	7	8	8	3	4
**27**	9	4	7	8	10	7	4
**28**	12	9	7	11	10	5	3
**29**	12	5	9	9	7	7	1
**30**	11	8	6	9	12	9	9
**31**	3	4	5	8	5	4	2
**32**	4	4	6	8	5	4	2
**33**	5	4	7	9	7	3	2
**34**	6	9	9	10	8	9	6
**35**	12	7	8	7	12	4	7
**36**	12	6	4	6	12	7	6

Abbreviations: Coping = Coping with adversity; Coach = Coachability; Concent = Concentration; Confiden = Confidence and achievement motivation; Goal = Goal setting/Mental preparation; Peak = Peaking under pressure; Free = Freedom from worry
